# Sildenafil attenuates pulmonary inflammation and fibrin deposition, mortality and right ventricular hypertrophy in neonatal hyperoxic lung injury

**DOI:** 10.1186/1465-9921-10-30

**Published:** 2009-04-29

**Authors:** Yvonne P de Visser, Frans J Walther, El Houari Laghmani, Hester Boersma, Arnoud van der Laarse, Gerry TM Wagenaar

**Affiliations:** 1 Department of Pediatrics, Division of Neonatology, Leiden University Medical Center, 2300 RC Leiden, the Netherlands; 2 Department of Cardiology, Leiden University Medical Center, 2300 RC Leiden, the Netherlands; 3 Department of Pediatrics, Los Angeles Biomedical Research Institute at Harbor-UCLA Medical Center, Torrance, CA 90502, USA

## Abstract

**Background:**

Phosphodiesterase-5 inhibition with sildenafil has been used to treat severe pulmonary hypertension and bronchopulmonary dysplasia (BPD), a chronic lung disease in very preterm infants who were mechanically ventilated for respiratory distress syndrome.

**Methods:**

Sildenafil treatment was investigated in 2 models of experimental BPD: a lethal neonatal model, in which rat pups were continuously exposed to hyperoxia and treated daily with sildenafil (50–150 mg/kg body weight/day; injected subcutaneously) and a neonatal lung injury-recovery model in which rat pups were exposed to hyperoxia for 9 days, followed by 9 days of recovery in room air and started sildenafil treatment on day 6 of hyperoxia exposure. Parameters investigated include survival, histopathology, fibrin deposition, alveolar vascular leakage, right ventricular hypertrophy, and differential mRNA expression in lung and heart tissue.

**Results:**

Prophylactic treatment with an optimal dose of sildenafil (2 × 50 mg/kg/day) significantly increased lung cGMP levels, prolonged median survival, reduced fibrin deposition, total protein content in bronchoalveolar lavage fluid, inflammation and septum thickness. Treatment with sildenafil partially corrected the differential mRNA expression of amphiregulin, plasminogen activator inhibitor-1, fibroblast growth factor receptor-4 and vascular endothelial growth factor receptor-2 in the lung and of brain and c-type natriuretic peptides and the natriuretic peptide receptors NPR-A, -B, and -C in the right ventricle. In the lethal and injury-recovery model we demonstrated improved alveolarization and angiogenesis by attenuating mean linear intercept and arteriolar wall thickness and increasing pulmonary blood vessel density, and right ventricular hypertrophy (RVH).

**Conclusion:**

Sildenafil treatment, started simultaneously with exposure to hyperoxia after birth, prolongs survival, increases pulmonary cGMP levels, reduces the pulmonary inflammatory response, fibrin deposition and RVH, and stimulates alveolarization. Initiation of sildenafil treatment after hyperoxic lung injury and continued during room air recovery improves alveolarization and restores pulmonary angiogenesis and RVH in experimental BPD.

## Introduction

Pharmacological and technical advances in neonatal intensive care medicine have greatly improved the survival and morbidity of premature infants. The preterm lung is highly susceptible to injury during resuscitation and mechanical ventilation and to pro-inflammatory mediators interfering with signaling required for normal late gestational lung development [1]. Preterm infants of < 30 weeks of gestation and a birth weight of < 1,200 g are at high risk for perinatal lung injury, that can progress to chronic lung disease (bronchopulmonary dysplasia, BPD). BPD is characterized by an arrest in alveolar and vascular lung development, complicated by inflammation, abnormal coagulation and fibrinolysis with intra-alveolar fibrin accumulation, oxidative stress, and at later stages by pulmonary hypertension and right ventricular hypertrophy [1,2].

Pharmacological treatment of BPD has relied upon systemic glucocorticoid administration, but has been refuted because of a higher incidence of neurological morbidity in long-term survivors. Theophylline, a non-selective phosphodiesterase (PDE) inhibitor, is widely used in neonatal intensive care to treat apnea of prematurity and wean preterm infants at risk for developing BPD from the ventilator, because it increases respiratory drive and has an immunomodulatory effect [3,4]. Since inflammation and unbalanced coagulation and fibrinolysis, leading to extravascular fibrin deposition in the lung, are two interrelated processes that play a pivotal role in the pathophysiology of inflammatory lung disease, we investigated whether the development of BPD can be interrupted by intervening in the vicious cycle of inflammation and coagulation. We have previously shown that anti-inflammatory agents, including the PDE4 inhibitors pentoxifylline, rolipram and piclamilast, and inhaled nitric oxide (NO) reduce fibrin deposition, pulmonary inflammation and prolong survival in rats with neonatal hyperoxic lung injury [5-7], a suitable *in vivo *model for experimental BPD [8]. PDEs exert their biological function by inactivating the intracellular messenger cAMP and cGMP by hydrolysis [9,10]. PDE5, a cGMP-specific inactivator, is expressed in smooth muscle cells, vascular endothelium, and platelets [9]. Inhibition of PDE5 increases intracellular cGMP levels. Inhibition of PDE5 promotes alveolar growth and angiogenesis, and attenuates inflammation and airway reactivity in animal models [11-15]. PDE5 inhibition also improves pulmonary vascular physiology in infants with persistent pulmonary hypertension, which may lead to prevention of right ventricular hypertrophy (RVH) [16,17].

To elucidate the role of PDE5 inhibition in the vicious circle of inflammation and coagulation in neonatal hyperoxic lung disease, we investigated the effect of sildenafil, a selective PDE5 inhibitor [18], using two different treatment strategies: a prophylactic strategy in a lethal model and a more clinically relevant strategy in which treatment was started after injury was induced in a non-lethal lung injury-recovery model. In the lethal model we show that sildenafil administration throughout the experimental period reduces inflammation, attenuates pulmonary fibrin deposition, improves alveolarization and angiogenesis, prevents RVH and prolongs survival of rat pups with hyperoxia-induced BPD. In the lung injury-recovery model we show that sildenafil treatment improves alveolarization and restores angiogenesis and RVH by reducing MLI, arteriolar wall thickness and increasing pulmonary vessel density and reducing right ventricular free wall thickness in rat pups with hyperoxia-induced BPD.

## Materials and methods

### Animals

The research protocol was approved by the Institutional Animal Care and Use Committee of the Leiden University Medical Center. Timed-pregnant Wistar rats were kept in a 12 h dark/light cycle and fed a standard chow diet (Special Diet Services, Witham, Essex, England) *ad libitum*. Breeding pairs were allowed access for one hour on the day female rats showed very specific sexual behaviour: lordosis, hopping and air-flapping. After a gestation of approximately 21^1/2 ^days pregnant rats were killed by decapitation (spontaneous birth occurs 22 days after conception) and pups were delivered by hysterectomy through a median abdominal incision to ensure that the delay in birth between the first and the last pup is only 5 min. Immediately after birth, pups were dried and stimulated. Pups from four litters were pooled and distributed over two experimental groups: the oxygen (O_2_) and the oxygen-sildenafil (sildenafil) group, and a room air-exposed (RA) control group. Litter size was 12 pups per litter in the experimental groups. Pups were kept in a transparent 50 × 50 × 70 cm Plexiglas chamber for 10 days or until death occurred (survival experiments). In this way influences of the birth process within and between litters can be avoided and exposure to hyperoxia can be started within 30 min after birth. Pups were fed by lactating foster dams, which were rotated daily to avoid oxygen toxicity. Foster dams were exposed to 100% oxygen for 24 h and next to room air for 48 h. The oxygen concentration was kept at 100% using a flow of 2.5 L/min. Oxygen concentrations were monitored daily with an oxygen sensor (Drägerwerk AG, Lübeck, Germany). Weight, evidence of disease, and mortality were also checked daily.

### Lethal neonatal hyperoxia model

In this model neonatal lung injury was induced by continuous exposure to 100% oxygen for 10 days. Starting on day 2, hyperoxia-exposed pups were injected daily subcutaneously with a 0.5 mL syringe (U-100 Micro-Fine insulin 29G syringe, Becton Dickinson, Franklin Lakes, NJ, USA) at the lower back. Pups received either 150 μL sildenafil citrate (a gift from Pfizer Limited, Sandwich, Kent, UK) in 0.9% saline or 150 μL 0.9% saline (age-matched control). In a pilot experiment in which rats were treated with 50–150 mg/kg/day sildenafil (25–75 mg/kg twice a day) under hyperoxia, we found that pups treated with 150 mg/kg/day sildenafil showed severe growth retardation and increased mortality. Therefore, experiments were performed with 50 and 100 mg/kg/day sildenafil. Separate experiments were performed for (1) survival studies, (2) collection of lung and heart tissue for fibrin deposition and RT-PCR, (3) histology, and (4) collection of bronchoalveolar lavage fluid.

### Neonatal lung injury-recovery model

The effect of sildenafil on lung injury and recovery was investigated by exposing newborn rat pups to hyperoxia for 9 days, followed by recovery in room air for 9 days. After 6 days of exposure to hyperoxia daily subcutaneous injections with 100 mg/kg/day sildenafil were started and continued throughout the 9-day recovery period in room air. Lung and heart tissue was collected for histology at the end of the 9-day hyperoxia period and after the 9-day recovery period in room air.

### Tissue preparation

Pups were anesthetized with an intraperitoneal injection of ketamine (25 mg/kg body weight; Nimatek, Eurovet Animal Health BV, Bladel, The Netherlands) and xylazine (50 mg/kg body weight; Rompun, Bayer, Leverkusen, Germany) on day 10. To avoid postmortem fibrin deposition in the lungs, heparin (100 units; Leo Pharma, Breda, The Netherlands) was injected intraperitoneally. After 5 min, pups were exsanguinated by transection of the abdominal blood vessels. The thoracic cavity was opened, and the lungs and heart were removed, snap-frozen in liquid nitrogen, and stored at -80°C until analysis by *real-time *RT-PCR, fibrin deposition or the cyclic GMP assay. For histology studies, the trachea was cannulated (Bioflow 0.6 mm intravenous catheter, Vygon, Veenendaal, The Netherlands), and the lungs and heart were fixed in situ via the trachea cannula with buffered formaldehyde (4% paraformaldehyde in PBS, pH 7.4) at 25 cm H_2_O pressure for 5 min. Lungs and hearts were removed, fixed (additionally) in formaldehyde for 24 h at 4°C, and embedded in paraffin after dehydration in a graded alcohol series and xylene. To quantify the degree of right ventricular hypertrophy (RVH), hearts were harvested, followed by the removal of left and right atria. Hereafter the right ventricular free wall (RV) was dissected, weighed separately from the interventricular septum (IVS) and left ventricle (LV), frozen immediately in liquid nitrogen, and stored at -80°C for *real time *RT-PCR. As an indicator of RVH the weight ratio RV/(LV + IVS) was calculated.

### Bronchoalveolar lavages

Pups were anesthetized with an intraperitoneal injection of ketamine and xylazine and injected intraperitoneally with heparin on day 10. A cannula (Bioflow 0.6 mm intravenous catheter, Vygon, Veenendaal, The Netherlands) was positioned in the trachea, and the pups were exsanguinated by transection of the abdominal blood vessels. Lungs were slowly lavaged two times with 500 μL 0.15 M NaCl, 1 mM EDTA (pH 8.0), without opening the thorax. Samples were pooled, stored temporarily at 4°C and centrifuged for 10 min at 5,000 rpm. Supernatants were stored at -20°C until further use.

### Histology

Paraffin sections (5 μm) were cut and mounted onto SuperFrost plus-coated slides (Menzel, Braunschweig, Germany). After deparaffinization, lung sections were stained with hematoxylin and eosin (HE) or with monoclonal anti-ED-1 antibody that specifically recognizes rat monocytes and macrophages [19], with polyclonal (rabbit) anti-myeloperoxidase (MPO) antibody [20], with monoclonal anti-alpha smooth muscle actin (ASMA) to visualize the pulmonary medial arterial walls or with polyclonal (rabbit) anti-von Willebrand Factor (vWF) as a marker for pulmonary blood vessels. Heart sections were stained with hematoxylin and eosin or with polyclonal (rabbit) anti-tenascin-C antibody, as an indicator for cardiac tissue damage [21]. For immunohistochemistry, sections were incubated with 0.3% H_2_O_2 _in methanol to block endogenous peroxidase activity. After a graded alcohol series, sections were boiled in 0.01 M sodium citrate (pH 6.0) for 10 min. Sections were incubated overnight with monoclonal anti-ED-1, polyclonal anti-MPO (Thermo Fisher Scientific, Fremont, CA, USA), monoclonal anti-ASMA (A2547, Sigma-Aldrich, St. Louis, MO, USA), polyclonal anti-vWF (A0082, Dako Cytomation, Glostrup, Denmark) or polyclonal anti-tenascin-C antibody (SC-20932, Santa Cruz Biotechnology, Santa Cruz, CA, USA), stained with EnVision-HRP (Dako, Glostrup, Denmark) using NovaRed (Vector, Burlingame, CA, USA) as chromogenic substrate, and counterstained briefly with hematoxylin. For morphometry of the lung, an eye piece reticle with a coherent system of 21 lines and 42 points (Weibel type II ocular micrometer; Paes, Zoeterwoude, The Netherlands) was used. Mean linear intercept (MLI), an indicator of mean alveolar diameter, was assessed in 10 non-overlapping fields at a 200× magnification in one HE-section for each animal. The density of ED-1 positive monocytes and macrophages or MPO-positive neutrophilic granulocytes was determined by counting the number of cells per field. Fields containing large blood vessels or bronchioli were excluded from the analysis. Results were expressed as cells per mm^2^. Per experimental animal 20 fields in one section were studied at a 400× magnification. Pulmonary alveolar septum thickness was assessed in HE-stained lung sections at a 400× magnification by averaging 100 measurements per 10 representative fields. Capillary density was assessed in lung sections stained for vWF at a 200× magnification by counting the number of vessels per field. At least 10 representative fields per experimental animal were investigated. Results were expressed as number of vessels per field. Pulmonary arteriolar wall thickness was assessed in lung sections stained for ASMA at a 1000× magnification by averaging at least 10 vessels with a diameter of less than 15 μm per animal. Fields containing large blood vessels or bronchioli were excluded from the analysis. Thickness of the right and left ventricular free walls and interventricular septum (IVS) was assessed in a transversal section taken halfway the long axis at a 40× magnification by averaging 6 measurements per structure. For morphometric studies in lung and heart at least 6 rat pups per experimental group were studied. Quantitative morphometry was performed by two independent researchers blinded to the treatment strategy.

### Fibrin detection assay

Fibrin deposition was detected in lung homogenates by Western blotting as described previously [8]. Tissue samples, dissolved in reducing sample buffer (10 mM Tris pH 7.5, 2% SDS, 5% glycerol, 5% β-mercaptoethanol, and 0.4 mg/mL bromophenol blue) were subjected to SDS-PAGE (7.5%; 5% stacking) and blotted onto PVDF membrane (Immobilon-P, Millipore, Bredford, MA, USA). The 56-kDa fibrin β-chains were detected with monoclonal 59D8 (Oklahoma Medical Research Foundation, Oklahoma City, OK, USA), which specifically recognizes β-fibrin [8,22], using ECL plus Western blotting detection system and Hyperfilm ECL (Amersham Biosciences, Arlington Heights, IL, USA). Exposures were quantified with a Bio-Rad GS-800 calibrated densitometer using the Quantity One, version 4.4.1 software package (Bio-Rad, Veenendaal, the Netherlands). Fibrin deposition was quantified in lungs of at least ten rats per experimental group using rat fibrin as a reference.

### Cyclic GMP assay

Lung tissue samples were homogenized in 10 volumes of 5% trichloroacetic acid (TCA) at 4°C. Samples were centrifuged at 1,500 g for 10 minutes. TCA was extracted from the supernatant by adding 5 volumes of water-saturated ether for 3 times. Residual ether was removed from the aqueous layer by heating at 70°C for 10 minutes. Cyclic GMP was detected in non-acetylated samples using a cyclic GMP EIA Kit (581021, Cayman Chemical Company, Ann Arbor, MI, USA) according to manufacturer's instructions.

### Real-time RT-PCR

Total RNA was isolated from lung and heart tissue homogenates using guanidium-phenol-chloroform extraction and isopropanol precipitation (RNA-Bee, Tel-Test Inc, Bio-Connect BV, Huissen, the Netherlands). The RNA sample was dissolved in RNase-free water and quantified spectrophotometrically. The integrity of the RNA was studied by gel electrophoresis on a 1% agarose gel, containing ethidium bromide. Samples did not show degradation of ribosomal RNA by visual inspection under ultraviolet light. First-strand cDNA synthesis was performed with the SuperScript Choice System (Life Technologies, Breda, the Netherlands) by oligo(dT)12–18 priming as described previously [8]. For *real-time *quantitative PCR, 1 μL of first-strand cDNA diluted 1:10 in RNase-free water was used in a total volume of 25 μL, containing 12.5 μL 2× SYBR Green PCR Master Mix (Applied Biosystems, Foster City, CA, USA) and 200 ng of each primer. Primers, designed with the Primer Express software package (Applied Biosystems), are listed in Table 1. Hyperoxia-induced lung injury induces alterations in inflammation, coagulation, fibrinolysis, alveolar enlargement, and edema. Therefore, we studied differential expression of key genes of these pathways, previously characterized in this rat model for experimental BPD [8], in lungs of pups exposed to room air, 100% oxygen, or 100% oxygen with 100 mg/kg/day sildenafil on postnatal day 10. PCR reactions consisting of 95°C for 10 min (1 cycle), 94°C for 15 s, and 60°C for 1 min (40 cycles), were performed on an ABI Prism 7900 HT Fast Real Time PCR system (Applied Biosystems) of the Leiden Genome Technology Center (Leiden, The Netherlands). Data were analyzed with the ABI Prism 7900 sequence detection system software (version 2.2) and quantified with the comparative threshold cycle method with β-actin as a housekeeping gene reference [23]. In a DNA array experiment we demonstrated that β-actin was not differentially expressed in lungs of hyperoxic rat pups compared to room air controls [8]. In addition β-actin was not differentially expressed in left and right ventricle in both control and experimental rat pups. In the heart samples mRNA expression in the RV was quantified relative to the expression in the LV and IVS.

**Table 1:** Sequences of oligonucleotides used as forward and reverse primers for real-time RT-PCR.

**Gene Product**	**Forward Primer**	**Reverse Primer**
Amphiregulin	5'-TTTCGCTGGCGCTCTCA-3'	5'-TTCCAACCCAGCTGCATAATG-3'
ANP	5'-CCAGGCCATATTGGAGCAAA-3'	5'-AGGTTCTTGAAATCCATCAGATCTG-3'
BNP	5'-GAAGCTGCTGGAGCTGATAAGAG-3'	5'-TGTAGGGCCTTGGTCCTTTG-3'
CNP	5'-AGGCAGCTGGTGGCAATC-3'	5'-GCGATCGGTCTCCCTTGAG-3'
FGFR4	5'-GTTGGCACGCAGCTCCTT-3'	5'-GCAGGACCTTGTCCAGAGCTT-3'
IL-6	5'-ATATGTTCTCAGGGAGATCTTGGAA-3'	5'-TGCATCATCGCTGTTCATACAA-3'
NPR-A	5'-CCTCCTGACGTCCCTAAATGTG-3'	5'-CCAGTGTGGAAAAGTGGTCTTG-3'
NPR-B	5'-TGAGCAAGCCACCCACTTC-3'	5'-CAGCGGGCCGCAGATATA-3'
NPR-C	5'-ACCAACAGCTCTCCTTGCAAA-3'	5'-AGGGCCCCCACAACAATT-3'
PAI-1	5'-AGCTGGGCATGACTGACATCT-3'	5'-GCTGCTCTTGGTCGGAAAGA-3'
TF	5'-CCCAGAAAGCATCACCAAGTG-3'	5'-TGCTCCACAATGATGAGTGTT-3'
VEGFR2	5'-CCACCCCAGAAATGTACCAAAC-3'	5'-AAAACGCGGGTCTCTGGTT-3'
β-actin	5'-TTCAACACCCCAGCCATGT-3'	5'-AGTGGTACGACCAGAGGCATACA-3'

### Protein assay

Total protein concentration was measured in bronchoalveolar lavage fluid (BALF) using the Dc protein assay (Bio-Rad, Veenendaal, the Netherlands), according to the manufacturer's instructions with bovine serum albumin, fraction V (Roche Diagnostics, Almere, The Netherlands) as a standard. The detection limit was 31 μg/mL.

### Statistical analysis

Values are expressed as mean ± SEM. Differences between groups (> 3) were analyzed with analysis of variance (ANOVA), followed by Tukey's multiple comparison test. For comparison of survival curves, Kaplan-Meier analysis followed by a log rank test was performed. Differences at *p *values < 0.05 were considered statistically significant.

## Results

### Lethal neonatal hyperoxia model

#### Fibrin deposition

**Figure 1:**
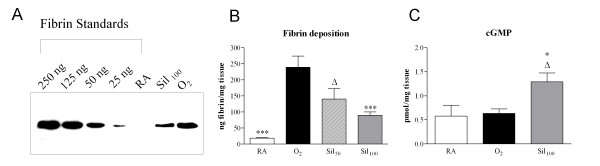
**Western blot analysis of fibrin deposition in lung homogenates of rat pups exposed to room air (RA), oxygen (O_2_) and O_2 _in combination with 100 mg/kg/day of sildenafil (Sil_100_) for 10 days (panel A)**. Panel B shows quantification of fibrin deposition in lung homogenates on day 10. Experimental groups include room air-exposed controls (RA, white bar), age-matched O_2_-exposed controls (O_2_, black bar) and sildenafil-treated rat pups (50 mg/kg/day: Sil_50_, striped bar; 100 mg/kg/day: Sil_100_, gray bar) under hyperoxia. Quantification of cyclic GMP in lung homogenates (panel C) in room air-exposed littermates (white bars), O_2_-exposed control pups (black bars) and 100 mg/kg/day sildenafil-treated pups (Sil_100_, gray bars). Data are expressed as mean ± SEM of at least 6 pups per experimental group. *p < 0.05 and ***p < 0.001 versus age-matched O_2_-exposed controls. ^Δ^*p *< 0.05 versus room air-exposed controls.

Because fibrin deposition is a sensitive marker for tissue damage in hyperoxia-induced neonatal lung disease, pulmonary fibrin deposition was studied in homogenates as a read-out for lung damage using Western blot analysis (Figure 1A) and quantified after treatment with two different sildenafil concentrations (50 and 100 mg/kg/day; Figure 1B). Fibrin deposition was at reference levels during normal neonatal pulmonary development on day 10 (18.4 ± 1.8 ng fibrin/mg tissue) and increased more than 13-fold to 239 ± 34.8 ng fibrin/mg tissue in lungs of pups exposed to 100% oxygen for 10 days (*p *< 0.001). Compared to oxygen-exposed controls, sildenafil treatment attenuated fibrin deposition in a concentration-dependent way by 62.5% to 89.8 ±10.3 ng fibrin/mg tissue for 100 mg/kg/day sildenafil (*p *< 0.05). Because 100 mg/kg/day of sildenafil was the most effective dose, additional experiments were limited to this dosage.

#### Cyclic GMP

To establish that sildenafil is a specific cyclic GMP dependent PDE inhibitor cyclic GMP levels were determined in lung tissue homogenates (Figure 1C). Exposure to hyperoxia for 10 days did not change cyclic GMP levels in lung homogenates compared to room air controls. Treatment with sildenafil resulted in a significant increase in cyclic GMP by 102% (*p *< 0.05) compared to oxygen-exposed controls.

#### Growth and survival

**Figure 2:**
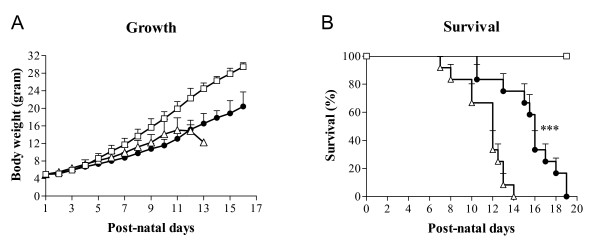
**Growth in sildenafil-treated rat pups (100 mg/kg/day, black circle), age-matched O_2_-exposed controls (open triangle) and room air exposed controls (open square) during the first 16 days after birth. Data are expressed as mean ± SEM (panel A)**. Kaplan-Meier survival curve of sildenafil-treated rat pups (black circle), age-matched O_2_-exposed controls (open triangle) and room air exposed controls (open square) during the first 19 days after birth (panel B). Data are expressed as percentage ± SEM of pups surviving at the observed time point. At least 12 pups per experimental group were studied. ***p < 0.001 for sildenafil-treated pups versus age-matched O_2_-exposed controls.

At birth, on postnatal day 1, mean body weight of the rat pups was 5.0 ± 0.18 g (Figure 2A). Body weight increased to approximately 8 grams on day 5 in oxygen exposed pups and room air controls. Hereafter, room air control pups grew slightly faster than oxygen-exposed pups. Growth of pups treated with 100 mg/kg/day sildenafil was not different from oxygen-exposed controls. Median survival of oxygen-exposed controls was 12 days and was prolonged with 4 days in pups treated with 100 mg/kg/day sildenafil and hyperoxia (Figure 2B; *p *< 0.001). After 13 days of oxygen exposure, 92% of the controls and only 25% of the sildenafil-treated pups had died. Room air-exposed pups did not show signs of illness or mortality during the first 4 weeks after birth.

#### Lung histology

**Figure 3:**
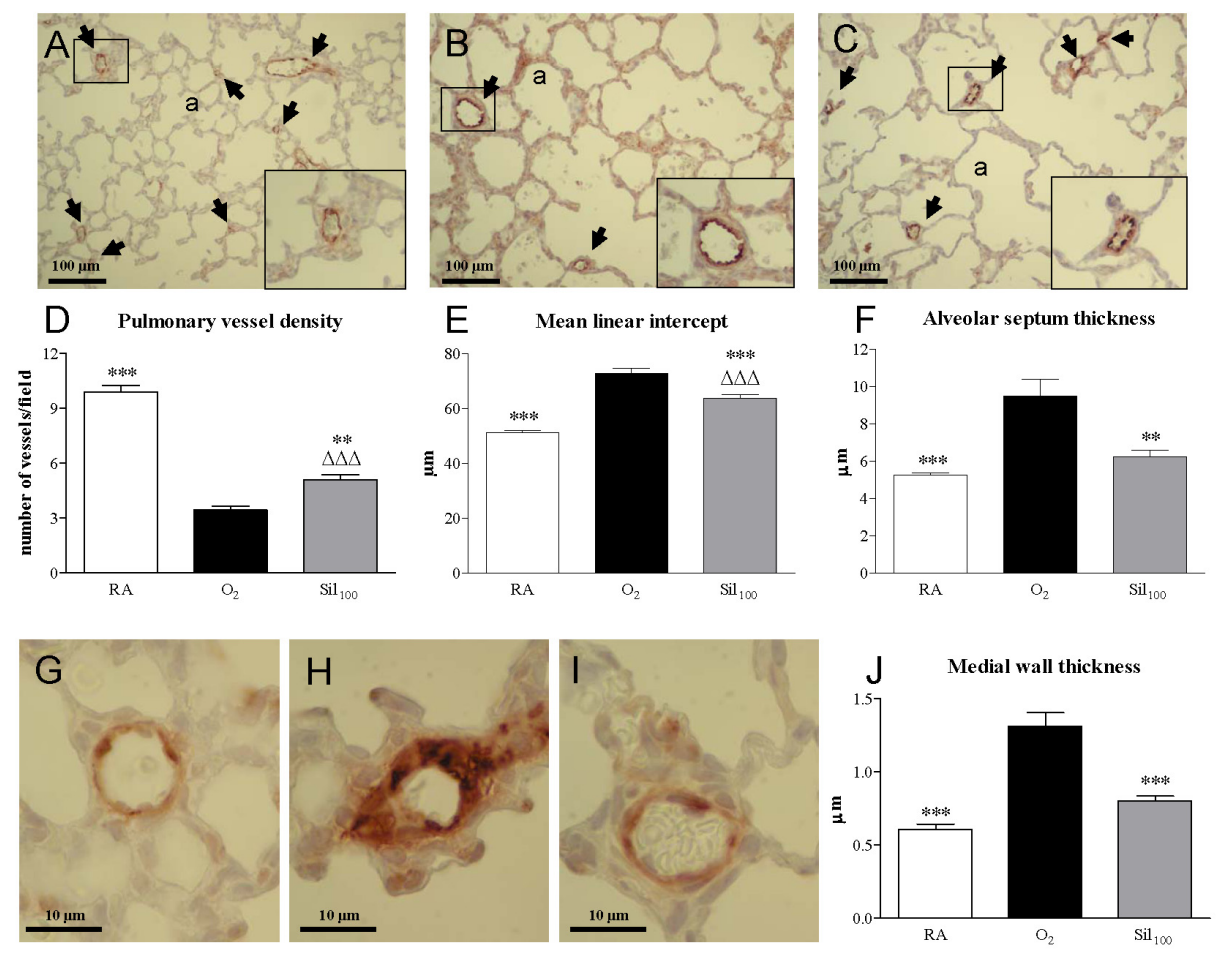
**Paraffin lung sections stained with polyclonal anti-vWF antibody (panels A-C) to visualize the endothelium of pulmonary vessels for the quantification of pulmonary vessel density (panel D) of room-air (RA, panel A) and O_2_-exposed controls (panel B), and age-matched pups treated with sildenafil (100 mg/kg/day) under hyperoxia (panel C) at 10 days of age**. Pictures were taken at a 200× magnification. Arrows in panels A-C indicate vWF-positive blood vessels. Quantification of pulmonary vessel density (panel D), mean linear intercept (panel E), alveolar septum thickness (panel F) and medial wall thickness (panel J) in room air-exposed littermates (white bars), O_2_-exposed control pups (black bars) and 100 mg/kg/day sildenafil-treated pups (Sil_100_, gray bars). Paraffin lung sections stained with monoclonal anti-ASMA antibody for the visualization of medial wall thickness in pulmonary arterioles (panels G-I) of room-air (RA, panel G) and O_2_-exposed controls (panel H), and age-matched pups treated with sildenafil (100 mg/kg/day) under hyperoxia (panel I) at 10 days of age. Pictures were taken at a 1000× magnification. The enlargements shown in the lower right parts of panels A, B and C are indicated in the boxed areas. Values are expressed as mean ± SEM in at least 6 different rat pups per group. a = alveolus **p < 0.01 and ***p < 0.001 versus age-matched O_2_-exposed controls. ^ΔΔΔ^*p *< 0.001 versus room air-exposed controls.

Lung development proceeds from the saccular stage at birth towards the alveolar stage on day 10 (Figure 3A). Oxygen exposure for 10 days resulted in edema, a reduction in pulmonary vessel density (Figure 3, panels B and D), a heterogeneous distribution of enlarged air-spaces with increased mean linear intercept (Figure 3E), which were surrounded by septa with increased thickness (Figure 3F) and an increase in pulmonary arteriolar medial wall thickness (Figure 3, panels H and J). Sildenafil treatment improved alveolarization and angiogenesis during hyperoxia exposure by increasing pulmonary vessel density (47.9%, *p *< 0.01; Figure 3, panels C and D), decreasing mean linear intercept (12.5%, *p *< 0.001; Figure 3E), thinning of alveolar septa (34.2%, *p *< 0.01; Figure 3F) and reducing arteriolar medial wall thickness (38.8%, *p *< 0.001; Figure 3, panels I and J) compared to oxygen exposure for 10 days.

**Figure 4:**
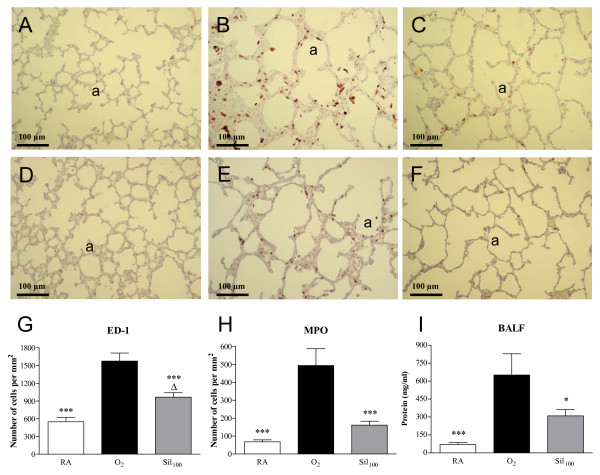
**Paraffin lung sections stained with monoclonal anti-ED-1 antibody (panels A-C) or polyclonal anti-MPO antibody (panels D-F) of room-air (RA, panels A and D) and O_2_-exposed controls (panels B and E), and age-matched pups treated with sildenafil (100 mg/kg/day) under hyperoxia (panels C and F) at 10 days of age**. Pictures were taken at a 200× magnification. Quantification of ED-1-positive monocytes and macrophages (panel G), MPO-positive neutrophilic granulocytes (panel H) and total protein concentration in bronchoalveolar lavage fluid (BALF; panel I) in room air-exposed littermates (white bars), O_2_-exposed control pups (black bars) and 100 mg/kg/day sildenafil-treated O_2_-exposed pups (Sil_100_, gray bars) for 10 days. Values are expressed as mean ± SEM in at least 6 different rat pups per group. Note the presence of large numbers of leukocytes, including macrophages and neutrophils in thickened septa and in the enlarged alveolar lumen in panels B and E in hyperoxia-exposed controls, and low numbers of pulmonary inflammatory cells after sildenafil treatment (panels C and F). a = alveolus. *p < 0.05 and ***p < 0.001 versus age-matched O_2_-exposed controls. ^Δ^*p *< 0.05 and versus room air-exposed controls.

Hyperoxia led to a massive inflammatory reaction, characterized by an overwhelming influx of inflammatory cells, including macrophages (Figure 4B) and neutrophils (Figure 4E), compared to room air-exposed controls (Figure 4, panels A and D). Resident ED-1-positive monocytes and macrophages were present at 548 cells per mm^2 ^in septa and alveoli of control lungs, whereas lungs of oxygen-exposed pups contained 2.9 times as many (*p *< 0.001; Figure 4G). Sildenafil treatment reduced the influx of ED-1-positive cells by 38.7% (*p *< 0.001; Figure 4, panels C and G) compared to oxygen-exposed controls. Resident MPO-positive neutrophils were present at 68 cells per mm^2 ^in septa and alveoli of control lungs, whereas lungs of oxygen-exposed pups contained 7.3 times as many (*p *< 0.001; Figure 4H). Sildenafil treatment reduced the influx of MPO-positive cells by 67.3% (*p *< 0.001; Figure 4, panels F and H) compared to oxygen-exposed controls.

#### Protein in bronchoalveolar lavage fluid

Total protein concentration in bronchoalveolar lavage fluid (BALF) was measured to establish the inhibitory effect of sildenafil on pulmonary edema by capillary-alveolar leakage (Figure 4I). Protein concentration on postnatal day 10 increased 9.4-fold after hyperoxia and had decreased by 52.5% after treatment with sildenafil (*p *< 0.05; hyperoxia versus sildenafil).

#### mRNA expression in lung tissue

**Figure 5:**
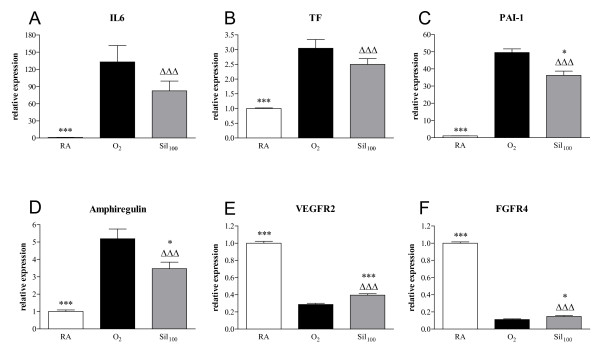
**Relative mRNA expression, determined with RT-PCR, of genes related to inflammation; interleukin-6 (IL-6; panel A), coagulation; tissue factor (TF; panel B), fibrinolysis; plasminogen activator inhibitor-1 (PAI-1; panel C) and alveolar growth; amphiregulin (panel D), vascular endothelial growth factor receptor-2 (VEGFR2; panel E) and fibroblast growth factor receptor-4 (FGFR4; panel F) in room air-exposed controls (RA, white bars), age-matched O_2_-exposed controls (O_2_, black bars) and sildenafil-treated rat pups (100 mg/kg/day [Sil_100_], gray bars) on day 10**. Data are expressed as mean ± SEM of 10 rat pups. *p < 0.05 and ***p < 0.001 versus age-matched O_2_-exposed controls.^ΔΔΔ^*p *< 0.001 versus room air-exposed controls.

Ten days of oxygen exposure resulted in an increase in mRNA expression of the pro-inflammatory cytokine IL-6 (133-fold; *p *< 0.001, Figure 5A), the procoagulant factor tissue factor (TF, 3.0-fold; *p *< 0.001, Figure 5B), the fibrinolytic factor plasminogen activator inhibitor-1 (PAI-1, 50-fold; *p *< 0.001, Figure 5C) and the growth factor amphiregulin (5.2-fold; *p *< 0.001, Figure 5D), and a decrease in the expression of vascular endothelial growth factor receptor-2 (VEGFR2, 3.5-fold; *p *< 0.001, Figure 5E) and fibroblast growth factor receptor-4 (FGFR4, 9.0-fold; *p *< 0.001, Figure 5F) in lungs of oxygen-exposed compared to room air-exposed pups. Treatment with 100 mg/kg/day sildenafil resulted in a reduction in PAI-1 (by 26.8%; *p *< 0.05, Figure 5C) and amphiregulin (by 33.3%; *p *< 0.05, Figure 5D) mRNA expression, whereas sildenafil treatment showed only a tendency towards lower IL-6 and TF mRNA expression compared to oxygen-exposed controls. In lung tissue of sildenafil-treated rat pups expression of VEGFR2 and FGFR4 mRNA was increased by 37.5% (*p *< 0.001) and by 32.6% (*p *< 0.05), respectively, compared to oxygen-exposed pups (Figure 5, panels E and F).

#### Right ventricular hypertrophy

**Figure 6:**
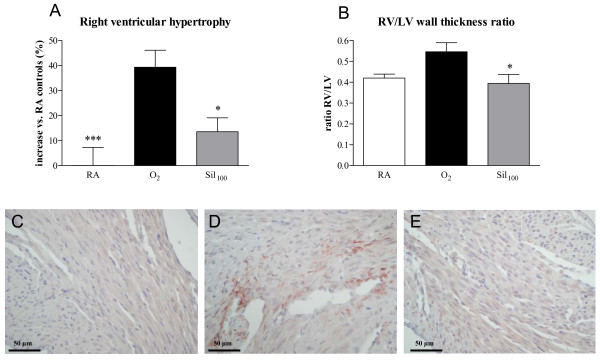
**Right ventricular hypertrophy is depicted as the increase in the ratio RV/(LV+IVS) compared to the room air control (panel A) and ventricular wall thickness, indicated as the RV/LV ratio (panel B) in room air-exposed controls (RA, white bars), age-matched O_2_-exposed controls (O_2_, black bars) and sildenafil-treated rat pups (100 mg/kg/day [Sil_100_], gray bars) under hyperoxia on day 10**. Cardiac characteristics are presented in table 2. Paraffin sections of the right ventricular wall stained with polyclonal tenascin C (panels C-E) of room-air (RA, panel C) and O_2_-exposed controls (panel D), and age-matched pups treated with sildenafil (100 mg/kg/day) under hyperoxia (panel E) at 10 days of age. Note the extravascular expression of tenascin C in the right ventricle in oxygen-exposed pups (panel D) and the absence of staining after treatment with sildenafil (panel E) and in room air controls (panel C). Pictures were taken at a 400× magnification.

Exposure to hyperoxia for 10 days resulted in RVH as demonstrated by a 1.4-fold increase in the weight ratio RV/(LV + IVS) compared to room air controls (*p *< 0.001; Table 2; Figure 6A). Treatment with sildenafil resulted in a significant regression of RVH (Figure 6A) and a decrease of the RV wall thickness by 26.8% compared to the oxygen-exposed controls (*p *< 0.05, Figure 6B). Extracellular expression of tenascin-C, a marker of myocardial overload, was visible in the RV only after exposure to hyperoxia. Tenascin-C expression was absent in room air exposed controls, as well as after treatment with sildenafil in experimental BPD (Figure 6, panels C-E).

**Table 2:** Cardiac characteristics

	RA	O_2_	Sil_100_
RV free wall thickness (μm)	240 ± 6	310 ± 34	197 ± 11*
LV free wall thickness (μm)	575 ± 13	568 ± 39	515 ± 34
IVS thickness (μm)	563 ± 67	568 ± 102	454 ± 62
RV/(LV+IVS)	0.302 ± 0.02***	0.412 ± 0.02	0.343 ± 0.01*

*** *p *< 0.001 and * *p *< 0.05 versus age-matched O_2 _exposed controls.

#### mRNA expression in the heart

**Figure 7:**
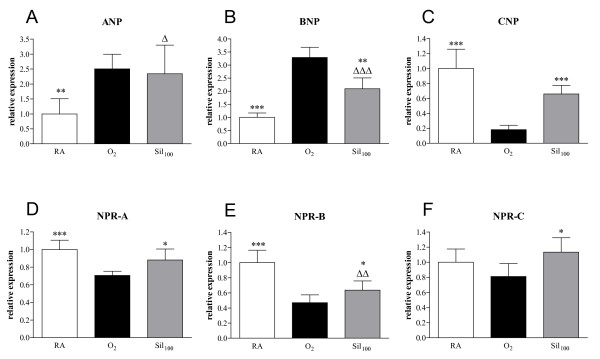
**mRNA expression in the right ventricle, relative to the expression in the left ventricle and interventricular septum, determined with RT-PCR, of atrial natriuretic peptide (ANP; panel A), brain natriuretic peptide (BNP; panel B), c-type natriuretic peptide (CNP; panel C), natriuretic peptide receptor (NPR) -A (panel D), NPR-B (panel E) and NPR-C (panel F) in room air-exposed controls (RA, white bars), age-matched O_2_-exposed controls (O_2_, black bars) and sildenafil-treated rat pups (100 mg/kg/day [Sil_100_], gray bars) under hyperoxia on day 10**. Data are expressed as mean ± SEM of 10 rat pups. *p < 0.05, **p < 0.01 and ***p < 0.001 versus age-matched O_2_-exposed controls.^Δ^*p *< 0.05, ^ΔΔ^*p *< 0.01 and ^ΔΔΔ^*p *< 0.001 versus room air-exposed controls.

Increased right ventricular mRNA expression was observed for the natriuretic peptides ANP (2.5-fold; *p *< 0.01, Figure 7A) and BNP (3.3-fold; *p *< 0.001, Figure 7B), whereas expression was decreased for CNP (5.5-fold; *p *< 0.001, Figure 7C) and for the natriuretic peptide receptors (NPR) -A (1.7-fold; *p *< 0.001, Figure 7D) and NPR-B (2.1-fold; *p *< 0.001, Figure 7E) after exposure to hyperoxia for 10 days compared to room air controls. Treatment with sildenafil decreased the expression of BNP (by 36.3%; *p *< 0.01) and increased the expression of CNP (by 267%; *p *< 0.001), NPR-A (by 24.7%; *p *< 0.05), NPR-B (by 35.7%; *p *< 0.05) and NPR-C (by 39.2%; *p *< 0.05, Figure 7F) compared to oxygen-exposed controls.

### Neonatal lung injury-recovery model

#### Lung histology

**Figure 8:**
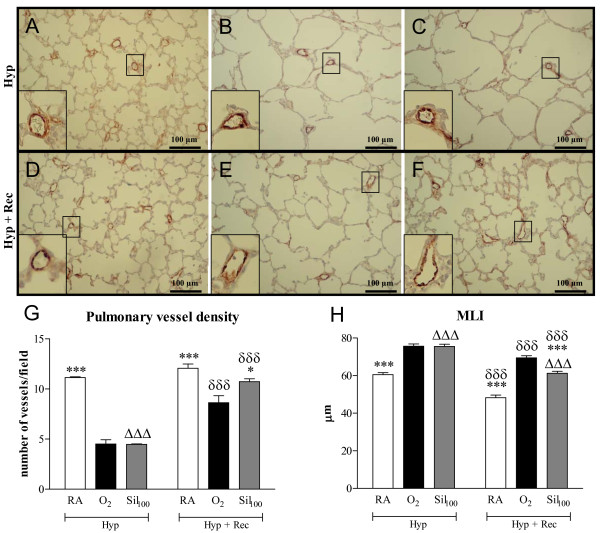
**Paraffin lung sections stained with polyclonal anti-vWF antibody (panels A-F) after hyperoxic injury for 9 days (panels A-C) and subsequent recovery in room air for 9 days (panels D-F) of room-air (RA, panel A), O_2_-exposed (panel B) and age-matched pups treated with sildenafil (100 mg/kg/day) under hyperoxia (panel C), and of RA (panel D), O_2_-exposed (panel E) and age-matched O_2_-exposed pups treated with sildenafil (100 mg/kg/day, panel F) after recovery**. Pictures were taken at a 200× magnification. Quantification of pulmonary vessel density (panel G) and mean linear intercept (panel H) after hyperoxic lung injury for 9 days (Hyp in panels G and H) and after recovery in room air for 9 days (Hyp + Rec in panels G and H) in room air-exposed (white bars), O_2_-exposed (black bars) and O_2_-exposed pups treated with 100 mg/kg/day sildenafil (Sil_100_, gray bars). The enlargements shown in the lower left parts of panels A-F are indicated in the boxed areas. *p < 0.05 and ***p < 0.001 versus age-matched O_2_-exposed controls.^ΔΔΔ^*p *< 0.001 versus room air-exposed controls. ^δδδ^*p *< 0.001 versus own treatment controls in hyperoxia period (hyp).

**Figure 9:**
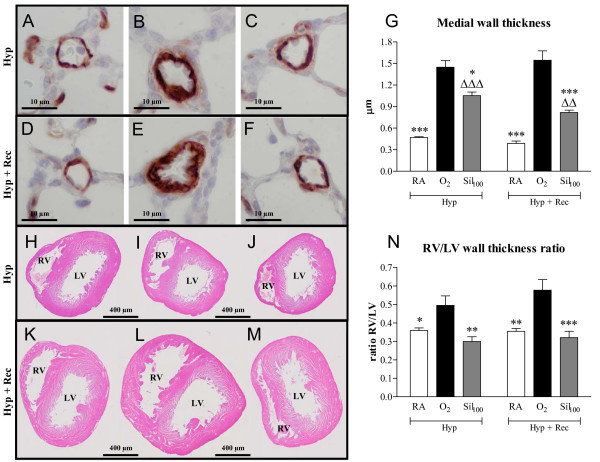
**Paraffin lung sections stained with monoclonal anti-ASMA antibody (panels A-F) and paraffin heart sections stained with HE (panels H-M) after hyperoxic injury for 9 days (panels A-C and H-J) and subsequent recovery in room air for 9 days (panels D-F and K-M) of room-air (RA, panels A and H), O_2_-exposed (panels B and I) and age-matched pups treated with sildenafil (100 mg/kg/day) under hyperoxia (panels C and J), and of RA (panels D and K), O_2_-exposed (panels E and L) and age-matched O_2_-exposed pups treated with sildenafil (100 mg/kg/day, panels F and M) after recovery**. Pictures were taken at a 1000× magnification (panels A-F) or at a 40× magnification (panels H-M). Quantification of pulmonary arteriolar medial wall thickness (panel G) and right ventricular hypertrophy (RV/LV wall thickness ratio, panel N) after hyperoxic lung injury for 9 days (Hyp in panels G and N) and after recovery in room air for 9 days (Hyp + Rec in panels G and N) in room air-exposed (white bars), O_2_-exposed (black bars) and O_2_-exposed pups treated with 100 mg/kg/day sildenafil (Sil_100_, gray bars). LV = left ventricle and RV = right ventricle. *p < 0.05, **p < 0.01 and ***p < 0.001 versus age-matched O_2_-exposed controls. ^ΔΔ^*p *< 0.01 and ^ΔΔΔ^*p *< 0.001 versus room air-exposed controls.

Continuous neonatal exposure to hyperoxia for 9 days resulted in a 2.5-fold reduction in blood vessel density (*p *< 0.001; Figure 8 panels B and G) and enlarged alveoli (Figure 8B), demonstrated by an increased MLI (*p *< 0.001, Figure 8H) and a 3.1-fold increase in medial wall thickness (*p *< 0.001; Figure 9, panels B and G) compared to room air controls. Sildenafil treatment during the last 3 days of the injurous hyperoxic period decreased medial wall thickness by 27.4% (*p *< 0.05 vs O_2_; Figure 9, panels C and G), but did not affect alveolar enlargement and blood vessel density (Figure 8, panels C, G and H). A recovery period of 9 days in room air after hyperoxia-induced lung injury (Figure 8E) reduced MLI (Figure 8H) and increased blood vessel density (Figure 8G), but alveoli continued to be enlarged (Figure 8E). Treatment with sildenafil restored blood vessel density (*p *< 0.05 vs O_2_; Figure 8, panels F and G) and reduced MLI by 11.8% (*p *< 0.001 vs O_2_, Figure 8H) compared to non-treated experimental BPD pups. However, medial wall thickness was only reduced in sildenafil-treated pups by 47% (*p *< 0.001; Figure 9, panels D-G) after a 9-day recovery period in room air.

Nine days of hyperoxic lung injury resulted in a 1.4-fold increase in the ratio RV/LV wall thickness, which was significantly reduced after sildenafil treatment for 3 days (42.2%; *p *< 0.001, Figure 9N). A recovery period of 9 days did not reduce RVH in the non-treated experimental BPD pups, but the RV/LV wall thickness ratio was completely restored after sildenafil treatment.

## Discussion

Prophylactic sildenafil therapy prolonged survival, improved lung histopathology, reduced RVH, and increased lung cGMP levels in neonatal rat pups exposed to continuous and prolonged hyperoxia, a suitable *in vivo *model for experimental BPD [8], by inhibiting inflammation, reducing capillary-alveolar protein leakage, alveolar septum thickness, and alveolar enlargement and by attenuating alveolar fibrin deposition in neonatal rat pups exposed to prolonged hyperoxia. Inhibition of lung inflammation was demonstrated by a reduction in the influx of inflammatory cells, including macrophages and neutrophilic granulocytes. Sildenafil therapy started after the initiation of hyperoxia-induced lung injury improved alveolarization and angiogenesis by attenuating alveolar enlargement and arteriolar medial wall thickness, and restoring pulmonary bloodvessel density and RVH in a lung injury-recovery model, demonstrating its therapeutic potential for treatment of BPD in the neonatal intensive care unit.

*In vitro *studies of lipopolysaccharide (LPS) mediated cytokine production in alveolar epithelial cells and *in vivo *studies on the influx of macrophages and neutrophils in a rat model of airway hyperreactivity have demonstrated the anti-inflammatory properties of PDE5 inhibition on pulmonary inflammatory processes [15,24]. Increased neo-vascularization in chicken chorioallantoic membranes has shown that sildenafil stimulation angiogenesis [25]. The improvement of alveolarization after sildenafil treatment in our study confirms, in part, the findings of Ladha et al, who investigated the effects of prophylactic sildenafil treatment in a similar rat model using quantitative histopathological techniques [14]. Lung injury in hyperoxia-exposed pups in this study was more severe as we used a different rat strain (Wistar instead of Sprague-Dawley rats, which are more resistant against hyperoxic lung injury), 100% instead of 95% oxygen and differences in the onset of lung injury.

We have previously shown that the specific inhibition of PDE4 with rolipram or piclamilast reduces alveolar fibrin deposition, inflammation and vascular alveolar leakage, and prolongs survival in rats with neonatal hyperoxic lung injury [6]. PDE4 inhibitors can exert their protective effect in inflammatory lung diseases by increasing intracellular cAMP levels [26]. PDEs belong to an enzyme family with 11 different members, designated PDE1-11, which exert their biological function by inactivating the intracellular messengers cAMP and/or cGMP by hydrolysis [26-28]. The beneficial effects of PDE5 inhibition by sildenafil on hyperoxia-induced lung injury may, at least in part, be due to higher intracellular cGMP levels as demonstrated by increased cGMP levels in lung homogenates (this study). In contrast to previous studies in which hyperoxic lung injury resulted in either increased [14,29] or decreased cGMP levels [30] we did not observe differences in cGMP levels in experimental BPD. This may be explained by differences in tissue source: plasma [14] versus lung tissue (this study) and the duration of the injurious hyperoxic response [30].

We have recently demonstrated that inhaled NO therapy improves lung pathology, reduces fibrin deposition and pulmonary inflammation, and prolongs survival in an animal model of BPD [7]. NO plays an important role in regulating pulmonary vascular tone and alveolar capillary development and in reducing inflammation in the developing lung [7,31,32]. Inhaled NO can exert its biological effects via the S-nitrosylation or via the NO-cGMP pathway [31,33,34]. The similarity of beneficial effects by inhaled NO and sildenafil treatment in experimental BPD suggests that the NO-cGMP pathway plays an important role in the pathogenesis of experimental BPD. Sildenafil-treated pups survived longer than pups treated with inhaled NO, but the effects of sildenafil treatment on pulmonary fibrin deposition and inflammation were less pronounced than the effects of inhaled NO. Intervention studies in hyperoxic lung injury with inhaled NO and (selective) PDE inhibitors have demonstrated less inflammation, but, incomplete restoration of lung development resulting in persistent enlarged alveoli [6,7,14,33]. Alveolar enlargement was accompanied by a downregulation of FGFR-4 which was partially restored after treatment with sildenafil. This confirms the observation that lungs of FGFR-3(-/-)/FGFR-4(-/-) mice are normal at birth, but have a complete block in alveogenesis and do not form secondary septa, demonstrating their cooperative function to promote the formation of alveoli [35].

NO stimulates the formation of cGMP in the endothelium and smooth muscle cells [14,36], whereas sildenafil protects cGMP from degradation by inhibiting PDE5 activity, but both modalities result in increased intracellular cGMP levels in these cells. Enhanced cGMP levels reduce pulmonary vascular resistance by relaxation of vascular smooth muscle cells and induce redistribution of pulmonary blood flow to ventilated lung regions, thereby preventing further lung injury [11,17,37]. Sildenafil and inhaled NO have both been used in term newborns with severe persistent pulmonary hypertension [16,17,37,38], a late complication of BPD. Early use of inhaled NO may improve the chances of survival without BPD in ventilated preterm infants [39], but data on sildenafil use in this group are not available. In addition, enhanced cGMP levels in endothelial cells improves angiogenesis and alveolarization via the vascular endothelial growth factor (VEGF)-NO-cGMP pathway [40,41]. Recombinant human VEGF treatment enhances alveolarization and vessel growth and improves lung structure in hyperoxia-induced neonatal lung injury [42,43]. On the contrary, VEGF blockade in newborn rats impairs alveolarization and vessel growth [44]. In experimental BPD in newborn rats alveolar enlargement and loss of lung capillaries are associated with decreased expression of lung VEGF and VEGF receptor-2 (VEGFR2) [44], whereas sildenafil improves alveolarization and angiogenesis [14], and reduces pulmonary fibrin deposition, inflammation and vascular alveolar leakage, resulting in prolonged survival in the present study. In lung injury-recovery models of experimental BPD alveoli are still enlarged after recovery in non-treated pups [42,44], but alveolarization and angiogenesis are almost completely restored after treatment with pro-angiogenic factors, such as VEGF [42,44] and sildenafil (this study). These results strongly suggest that sildenafil treatment of preterm infants may reverse the arrest in lung development which is typical for those developing BPD.

Sildenafil treatment improved hyperoxia-induced RVH in experimental BPD (this study and [14]), reduced extracellular tenascin-C expression in the RV, a marker that is upregulated under myocardial stress conditions [45,46], and reduced the thickness of the RV. The beneficial effect of sildenafil on the heart can be explained either directly or indirectly by a reduction of pulmonary hypertension resulting in reduced RVH. This is supported by a sildenafil-induced reduction in pulmonary arteriolar wall thickness (this study) and by similar beneficial effects of PDE5-inhibitors in experimental models of lung disease, including monocrotaline-induced pulmonary hypertension and bleomycin-induced pulmonary fibrosis [47-49]. A direct beneficial effect of sildenafil is supported by an induction of PDE5 in the myocardium of the hypertrophied LV or RV in patient material and in the RV after monocrotaline-induced RVH in rats [50]. In addition, Nagendran et al. have demonstrated that sildenafil treatment restored the upregulated cGMP-PDE activity in RV of rats with monocrotaline-induced pulmonary artery hypertension and increased RV contractility of these rats.

The natriuretic peptides atrial natriuretic peptide (ANP) and brain natriuretic peptide (BNP) are synthesized and released in response to atrial pressure and ventricular overload, respectively, and their plasma concentrations are related to ventricular dysfunction and severity of cardiac pathology [51,52]. Occupation of the natriuretic peptide receptor (NPR) -A, activated by ANP, BNP and DNP, and NPR-B, which is specific to CNP, induces cellular responses via activation of particulate guanylate cyclase, in contrast to soluble guanylate cyclase that is activated by NO, thereby elevating the intracellular levels of cGMP [53,54]. As markers for RVH we studied the differential expression of ANP, BNP, CNP and the natriuretic peptide receptors NPR-A, NPR-B and NPR-C at the mRNA level. Hyperoxia-induced RVH resulted in reduced expression of the guanylate cyclase-coupled natriuretic peptide receptors NPR-A and NPR-B in cardiomyocytes. Signaling after activation of these receptors by natriuretic peptides is mediated by cGMP [54]. This suggests that the intracellular cGMP concentration in the hypertrophic RV cardiomyocyte is not only lowered by increased PDE5 expression, but may also be reduced due to decreased levels of NPR-A and NPR-B, which can be restored, at least in part, by sildenafil treatment.

## Conclusion

The beneficial effects of sildenafil on alveolarization, lung inflammation and extravascular fibrin deposition, right ventricular hypertrophy and survival in neonatal rats with hyperoxia-induced lung injury emphasise the potential of phosphodiesterase 5 inhibitors as treatment for bronchopulmonary dysplasia in premature infants.

## Abbreviations

ANP: atrial natriuretic peptide; ASMA: alpha smooth muscle actin; BNP: brain natriuretic peptide; BALF: bronchoalveolar lavage fluid; BPD: bronchopulmonary dysplasia; cAMP: cyclic adenosine monophosphate; cGMP: cyclic guanosine monophosphate; CNP: c-type natriuretic peptide; FGFR4: fibroblast growth factor receptor-4; IL: interleukin; IVS: interventricular septum; LV: left ventricle; MLI: mean linear intercept; MPO: myeloperoxidase; NO: nitric oxide; NPR: natriuretic peptide receptor; O_2_: oxygen; PAI-1: plasminogen activator inhibitor-1; PDE: phosphodiesterase; RA: room air; RT-PCR: reverse transcriptase polymerase chain reaction; RV: right ventricular free wall; TF: tissue factor; VEGFR2: vascular endothelial growth factor (VEGF) receptor-2; vWF: Von Willebrand Factor.

## Competing interests

The authors declare that they have no competing interests.

## Authors' contributions

YPV, EHL and HB carried out the experimental studies. YPV drafted the manuscript. GTMW, FJW and AL designed the experimental setup and provided intellectual input in the manuscript preparation. GTMW supervised the work.
